# Roles of hsa-miR-12462 and SLC9A1 in acute myeloid leukemia

**DOI:** 10.1186/s13045-020-00935-w

**Published:** 2020-07-23

**Authors:** Yan Jia, Wei Liu, Hui-En Zhan, Xiao-Ping Yi, Hui Liang, Qi-Li Zheng, Xin-Ya Jiang, Hai-Yan Zhou, Liang Zhao, Xie-Lan Zhao, Hui Zeng

**Affiliations:** 1 Department of Hematology, Xiangya Hospital, Central South University, Changsha, Hunan China; 2 Department of Hematology, The First Affiliated Hospital of Jinan University, Guangzhou, Guangdong China; 3 Department of Radiology, Xiangya Hospital, Central South University, Changsha, Hunan China; 4 Department of Pathology, Xiangya Hospital, Central South University, Changsha, Hunan China

**Keywords:** miRNA, Acute myeloid leukemia, SLC9A1, RNA sequencing

## Abstract

MicroRNAs (miRNAs) play important roles in cell proliferation, differentiation, and survival and may be useful for acute myeloid leukemia (AML) diagnosis and prognosis. In this study, we defined a novel miRNA, hsa-miR-12462, through small RNA sequencing of the bone marrow (BM) cells from 128 AML patients. Overexpression of hsa-miR-12462 in AML cells (U937 and HL-60) significantly decreased their growth rate when compared with those of the wild-type and MOCK controls. In a xenograft mouse model, tumor weight and size in the mice bearing the U937 cells with hsa-miR-12462 overexpression were significantly reduced when compared with those bearing the mock cells. The AML cells overexpressing hsa-miR-12462 had increased sensitivity to cytarabine chemotherapy. Combining the data from the MiRDB, an online microRNA database (http://mirdb.org), with the RNA-sequencing results, *SLC9A1* was predicted to be one of the targets of hsa-miR-12462. hsa-miR-12462 was further confirmed to bind exclusively to the 3′UTR of *SLC9A1* in U937 cells, leading to downregulation of SLC9A1. In summary, a higher level of hsa-miR-12462 in AML cells is associated with increased sensitivity to cytarabine chemotherapy via downregulation of *SLC9A1.*

To the Editor,

MicroRNAs (miRNAs) play important roles in cell proliferation, differentiation, and survival and may be useful for acute myeloid leukemia (AML) diagnosis and prognosis [1–4]. In this study, we defined a novel miRNA, hsa-miR-12462, through small RNA sequencing of the bone marrow (BM) cells from 128 newly diagnosed subjects with AML (Supplementary Table1-2). Based on 2016 World Health Organization (WHO) criteria, all subjects were grouped into 2 cohorts: (1) those achieving a complete remission (CR) with conventional induction chemotherapy and remaining in CR ≥ 6 months and (2) those not achieving CR after 2 courses of standard induction chemotherapy (refractory) or relapsed in < 6 months after CR (relapsed) [5]. Small RNA sequencing of these samples revealed different miRNA expression profiles between CR and refractory/relapsed (RR) AML patients [6]. One miRNA showed the highest differential expression pattern in this analysis. This miRNA has never been reported in the literature. We named this miRNA as hsa-miR-12462 (Figure S1A). Next, we explored the biological activity of hsa-miR-12462 by overexpressing it in AML cells using a lentiviral vector (Figure S1B). The growth rate of the hsa-miR-12462 overexpressing cells was significantly decreased when compared with those of the wild-type and MOCK controls in both U937 and HL-60 cells (Fig. 1a, Figure S1C). U937 cells were confirmed using an EdU incorporation assay (Figure S1D-E).

**Fig. 1:**
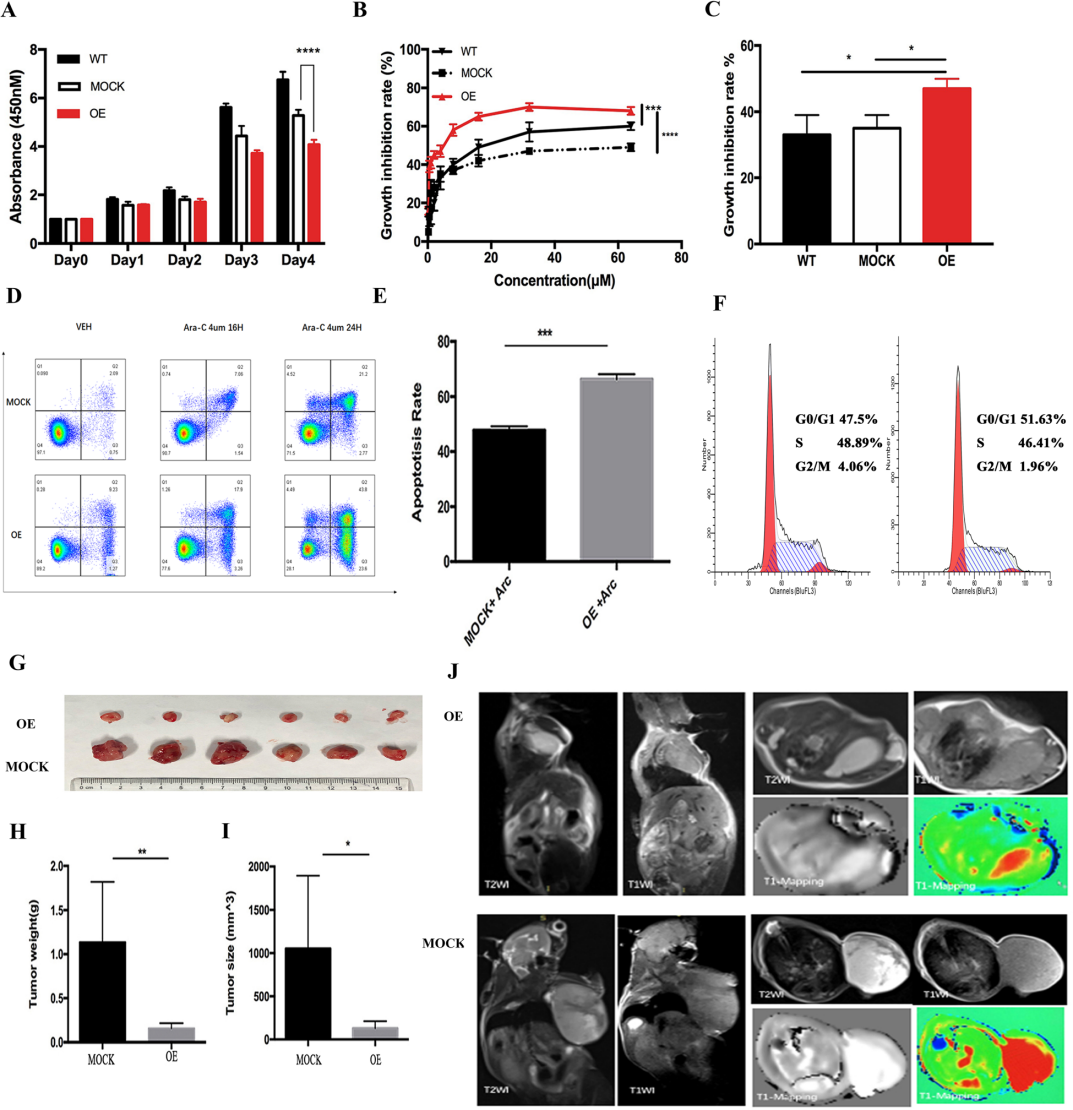
Overexpression of hsa-miR-12462 inhibits the growth of U937 cells and increases cytarabine sensitivity. **a** Growth rates of U937 wild-type, MOCK-infected, and overexpressing cells (OE vs. MOCK *P* < 0.0001). **b** Growth inhibition of wild-type, MOCK-infected, and overexpressing U937 AML cells treated with cytarabine (WT vs. OE *P* < 0.0001; MOCK vs. OE *P* < 0.0001). **c** Growth inhibition of wild-type, MOCK-infected, and overexpressing U937 cells treated with cytarabine at the IC_50_ of 4 μM (WT vs. OE *P* < 0.005; MOCK vs. OE *P* < 0.005). **d**, **e** Percent apoptosis of MOCK-infected and overexpressing U937 AML cells treated with cytarabine by FACS analysis (**e**, OE vs. MOCK *P* < 0.0001). **f** Representative histograms of cell cycle phases in MOCK-infected and overexpressing U937 cells by FACS analysis. **g** The tumor tissues from the xenograft mouse model injected with wild-type (MOCK) and hsa-miR-12462 overexpressing U937 cells (OE). **h**, **i** Tumor weights (**h**) and sizes (**i**) from mice injected with wild-type and overexpressing U937 cells (MOCK vs. OE 1.11380 ± 0.27820 g vs. 0.16000 ± 0.02266 g, *n* = 6, *P* = 0.0057). **j** Axial anatomic image of mice injected with overexpressing and wild-type U937 cells obtained with 3.0-T MRI. FOV = 64 mm

Treatment of AML cells with cytarabine for up to 48 h resulted in a lower proliferation of the hsa-miR-12462 overexpressing cells when compared with that of the controls (Fig. 1b, c, Figure S1F-G). The apoptosis rate of the hsa-miR-12462 overexpressing cells was significantly higher than that of the control (Fig. 1d, e, Figure S1I,L) as well. These differences were not seen in U937 cells (Figure S1H,J) or HL-60 cells (Figure S1K) treated with doxorubicin. We hypothesized that these different responses might reflect the effects of hsa-miR-12462 on the cell cycle. Cell cycle analysis revealed that a greater proportion of hsa-miR-12462-expressing cells were adjusted in G_0_/G_1_ and S-phase when compared with MOCK-transfected cells (Fig. 1f, Figure S1M).

We further studied the biological behavior of the hsa-miR-12462 overexpressing U937 cells in a subcutaneous xenograft mouse model (Fig. 1g). Tumor weight and size in the overexpressing cohort were decreased when compared with those of the MOCK-transfected cohort (Fig. 1h, i). (MOCK vs. OE: *P* = 0.0057). These differences were further confirmed by both magnetic resonance imaging (MRI; Fig. 1j) and histopathology (Figure S1N-P).

Through RNA-sequencing analysis of hsa-miR-12462 overexpressing and MOCK-transfected U937 cells, 306 genes were identified with differential expressions (Fig. 2a, S2A). Gene Ontology (GO) and Kyoto Encyclopedia of Genes and Genomes (KEGG) pathway enrichment analyses were done as well (Figure S2B-C). Enrichment analysis of the KEGG pathway indicates the involvement of the cAMP signaling pathway (Figure S2C) [7]. In addition, *CCNE1*, *E2F4*, and *TP53* involved in cell cycle regulation were found significantly downregulated in the RNA-sequencing analysis (Figure S2A). Q-RT-PCR analysis of these genes confirmed the results from the RNA-sequencing analysis (Fig. 2b–d). Combining the data from the MiRDB, an online microRNA database (http://mirdb.org) [8], with the RNA-sequencing results, 15 genes were found to share predicted targets (Fig. 2e, f) including *SLC9A1*, *ARRB1*, and *CHRNA6* (Fig. 2g). *SLC9A1* (NHE1), the most common isomer in the Na^+^/H^+^ exchanger family [9], is important in cell transformation [10]. β-arrestins (ARRBs) participate in mediating tumor proliferation and inflammation-induced cancer development [11], whereas nicotinic acetylcholine receptors (CHRNs) are important regulators of tobacco-induced carcinogenesis [12]. The mRNA and protein levels of *SLC9A1*, *ARRB1*, *and CHRNA6* were inhibited by overexpression of hsa-miR-12462 (Fig. 2h, i, S2D-E). Using a luciferase 3′UTR reporter assay, we found that hsa-miR-12462 bound exclusively to the 3′UTR of *SLC9A1* in U937 cells (Fig. 2j; S2F-G). In summary, a higher level of hsa-miR-12462 in AML cells is associated with increased sensitivity to cytarabine chemotherapy via downregulation of *SLC9A1.*

**Fig. 2:**
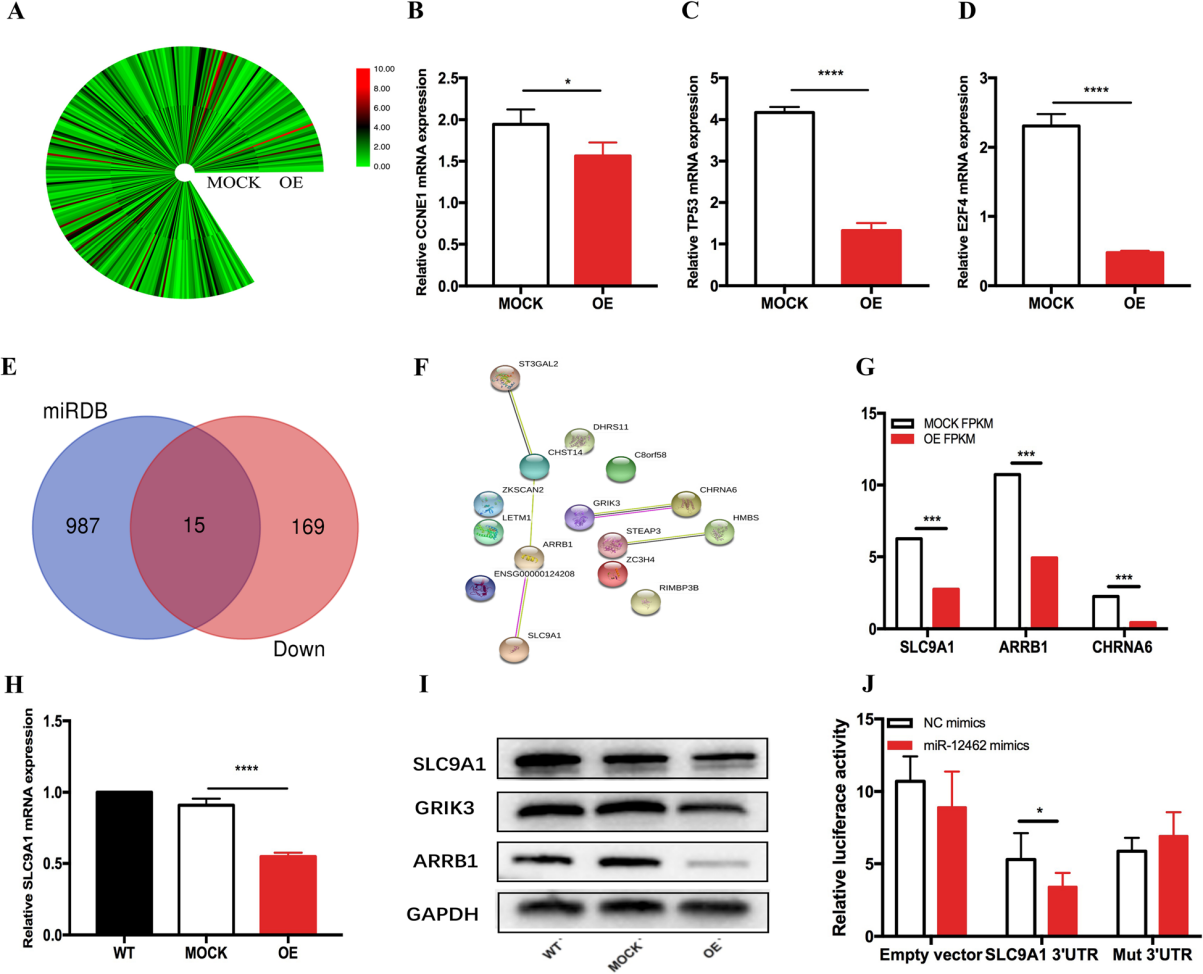
hsa-miR-12462 acts via downregulation of *SLC9A1*. **a** Overview of mRNAs in OE and MOCK U937 cells. **b**–**d***CCNE1* (**b**), *TP53* (**c**), and *E2F4* (**d**) transcript expression in wild-type, MOCK-infected, and overexpressing U937 AML cells is shown (**b**, OE vs. MOCK *P* = 0.0001; **c**, OE vs. MOCK *P* = 0.0001; **d**, OE vs. MOCK *P* = 0.0329). Primers were CCNE1 F: AGC GGT AAG AAG CAG AGC AG, R: TTT GAT GCC ATC CAC AGA AA; TP53 F: CCT CAG CAT CTT ATC CGA GTG G, R: TGG ATG GTG GTA CAG TCA GAG C; and E2F4 F: GAG TGG TCC CAT TGA GGT TC, R: GGC AGA GGT GGA GGT GTA G. **e** Venn diagram of differentially expressed genes as determined by RNA-sequencing analysis. **f** Protein-protein interaction network of 15 target genes. **g** Expression of *SLC9A1*, *ARRB1*, and *CHRNA6* in U937 MOCK-infected and overexpressing U937 cells is shown (MOCK vs. OE *P* < 0.0001). **h** Expression of *SLC9A1* transcript in wild-type, MOCK-infected, and overexpressing U937 cells is shown. **i** Expression of *SLC9A1*, *ARRB1*, and *CHRNA6* in U937 wild-type, MOCK-infected, and overexpressing cells by western blot. GAPDH is used as a control. **j***SLC9A1* is a direct target of hsa-miR-12462 confirmed by luciferase activity. Luciferase constructs containing the 3′UTR of *SLC9A1* or 3′UTR with point mutations

## Supplementary information

**Additional file 1: Table S1.** Patient information. **Table S2.** Demographic and AML - related features of the expression level of hsa-miR-12462

**Additional file 2: Figure S1.** Hsa-miR-12462 inhibits growth of AML both in cell lines and animal models. A.Schematic diagram of the secondary structure of mature hsa-miR-12462.Structure was predicted by MFOLD. B. hsa-miR-12462 transcript expression in Wild-type, MOCK-infected, and Over-expressing U937 cells (OE *vs*. MOCK P = 0.0005). C. Growth rates of HL-60 Wild-type, MOCK-infected and Over-expressing cells (OE *vs*. MOCK P < 0.0001). D-E. Cell growth of MOCK and Over-expressing U937 OE *vs*. MOCK P = 0.0004) cells as measured by EdU. F. Growth inhibition of Wild-type, MOCK infected and Over-expressing HL-60 cells treated with cytarabine(OE *vs*. MOCK P=0.0428). G. Growth inhibition of Wild-type, MOCK-infected, and Over-expressing HL-60 cells treated with cytarabine at the IC_50_ = 2.9 μM (MOCK *vs*. OE P = 0.0042). H . Growth inhibition rates of Wild-type, MOCK-infected and Over-expressing U937 cells treated with doxorubicin IC_50_ = 6 μM (OE *vs*. MOCK *vs.* WT P > 0.05). I,L. Percent apoptosis of MOCK infected and Over-expressing HL-60 cells treated with cytarabine by FACS analysis (L, OE *vs*. MOCK P =0.0377). J,K: Apoptosis percentage of MOCK-infected and Over-expressing U937(J) and HL-60(K) cells treated with doxorubicin by FACS analysis.( J:OE *vs*. MOCK P > 0.05.K:OE *vs*. MOCK P > 0.05) M. Quantification of cell-cycle phases in MOCK-infected and over-expressing U937 cells by FACS analysis. N. Hematoxylin and eosin (HE) staining and immune histochemistry analyses of hsa-miR-12462 OE and MOCK tumor sections. HE staining magnification: ×20. MPO staining magnification: ×20. Ki-67 staining magnification: ×20.O-P. Percentage of myeloperoxidase (MPO) and Ki-67 expression in OE and MOCK U937 cells (MOCK *vs*. OE: O, P<0.0001. P, P<0.0001)

**Additional file 3: Figure S2.** The downstream targets of hsa-miR-12462. A. Differential expression of selected genes in hsa-miR-12462 OE and MOCK U937cells. B-C. (B) GO and (C) KEGG pathway enrichment analysis of differentially expressed genes after RNA-sequencing in U937 OE/MOCK cells. D-E. Transcript expression of CHRNA6 (D) and ARRB1 (E) in wild-type, MOCK-infected and OE U937 cells is shown. F-G. Luciferase reporter assay showing the 3'-UTR segments of (F) *ARRB1* and (G) *CHRNA6* do not contain hsa-miR-12462 binding sites. Error bars indicate ± SD; n=3 each. P values were obtained by the two-tailed Student t-test.

**Additional file 4.** Detailed materials and methods

## Data Availability

All supporting data are included in the manuscript and supplemental files. Additional data are available upon reasonable request to the corresponding author.

## Abbreviations

AML: Acute myeloid leukemia
*SLC9A1*: Solute carrier family 9 member A1
cAMP: Cyclic adenosine monophosphate
*CCNE1*: CyclinE1
*E2F2*: *E2F* transcription factor 2
*ARRB1*: Arrestin beta-1
*CHRNA6*: *C*holinergic receptor, nicotinic, alpha 6
