# Supplementary material for: Roles of hsa-miR-12462 and SLC9A1 in acute myeloid leukemia
Source: J Hematol Oncol. 2020 Jul 23;13:101. doi: 10.1186/s13045-020-00935-w (PMC7376648; doi:10.1186/s13045-020-00935-w)
Supplement: Supplementary file 1 — Additional file 1: Table S1. Patient information. Table S2. Demographic and AML - related features of the expression level of hsa-miR-12462 [file 13045_2020_935_MOESM1_ESM.docx]

**Table 1. Patient information.**

| Patient characteristic | AML-RR (n=38) | AML-CR (n=90) | P |
| --- | --- | --- | --- |
| Age(Mean±SD) | 47. ±14.93 | 44±15.62 | 0.32 |
| Sex |  |  |  |
| Male | 20 (52.6) | 36 (40) | 0.19 |
| Female | 18 (47.4) | 54 (60) |  |
| BM balsts(Mean±SD, %) | 46.61±13.81 | 44.3±14.49 | 0.41 |
| Cyto-genetic risk stratification |  |  |  |
| Good | 5 (13.2) | 20(22.2) | 0.00 |
| Intermediate | 12 (31.6) | 47(52.2) |  |
| Poor | 21 (55.2) | 23(25.6) |  |

**Table 2. Demographic and AML - related features of the expression level of hsa-miR-12462**

|  | High miR-12462 level | Low miR-12462 level | χ2 | P |
| --- | --- | --- | --- | --- |
| Age |  |  |  |  |
| <60 | 38（29.7） | 37（28.9） | 0.03 | 0.86 |
| ≥60 | 26（20.3） | 27（21.1） |  |  |
| Gender |  |  |  |  |
| Male | 29（22.7） | 27（21.1） | 0.13 | 0.72 |
| Female | 35（27.3） | 37（28.9） |  |  |
| AML condition |  |  |  |  |
| CR | 54（42.2） | 36（28.1） | 12.13 | 0.00 |
| R/R | 10（7.8） | 28（21.9） |  |  |
| BM balsts(Mean±SD, %) | 39.75±13.28 | 64.83±21.01 |  | 0.00 |
| Cyto-genetic risk stratification |  |  |  |  |
