# Supplementary figures and images for: Roles of hsa-miR-12462 and SLC9A1 in acute myeloid leukemia
Source: J Hematol Oncol. 2020 Jul 23;13:101. doi: 10.1186/s13045-020-00935-w (PMC7376648; doi:10.1186/s13045-020-00935-w)

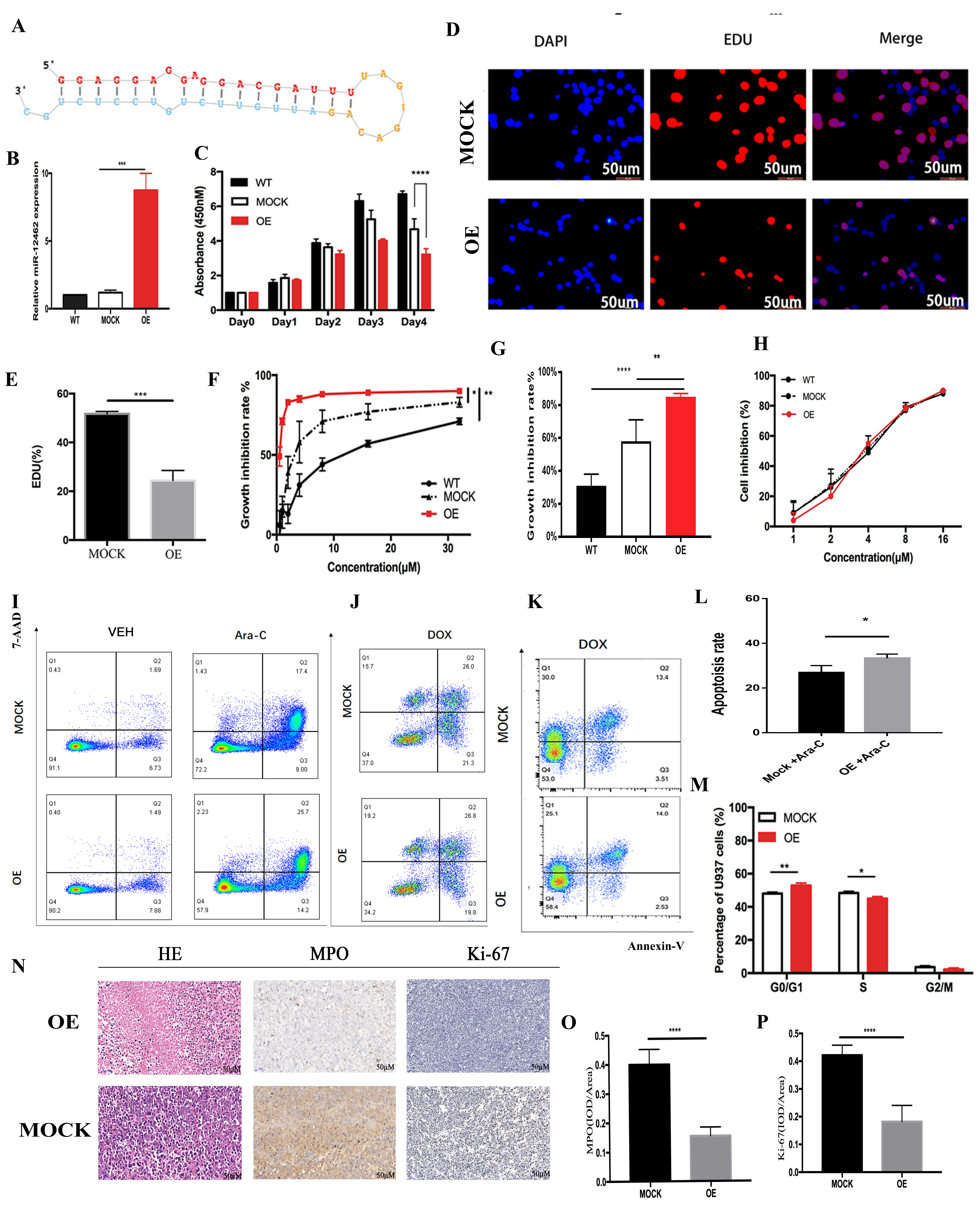

Supplement: Supplementary file 2 — Additional file 2: Figure S1. Hsa-miR-12462 inhibits growth of AML both in cell lines and animal models. A.Schematic diagram of the secondary structure of mature hsa-miR-12462.Structure was predicted by MFOLD. B. hsa-miR-12462 transcript expression in Wild-type, MOCK-infected, and Over-expressing U937 cells (OE vs. MOCK P = 0.0005). C. Growth rates of HL-60 Wild-type, MOCK-infected and Over-expressing cells (OE vs. MOCK P < 0.0001). D-E. Cell growth of MOCK and Over-expressing U937 OE vs. MOCK P = 0.0004) cells as measured by EdU. F. Growth inhibition of Wild-type, MOCK infected and Over-expressing HL-60 cells treated with cytarabine(OE vs. MOCK P=0.0428). G. Growth inhibition of Wild-type, MOCK-infected, and Over-expressing HL-60 cells treated with cytarabine at the IC50 = 2.9 μM (MOCK vs. OE P = 0.0042). H . Growth inhibition rates of Wild-type, MOCK-infected and Over-expressing U937 cells treated with doxorubicin IC50 = 6 μM (OE vs. MOCK vs. WT P > 0.05). I,L. Percent apoptosis of MOCK infected and Over-expressing HL-60 cells treated with cytarabine by FACS analysis (L, OE vs. MOCK P =0.0377). J,K: Apoptosis percentage of MOCK-infected and Over-expressing U937(J) and HL-60(K) cells treated with doxorubicin by FACS analysis.( J:OE vs. MOCK P > 0.05.K:OE vs. MOCK P > 0.05) M. Quantification of cell-cycle phases in MOCK-infected and over-expressing U937 cells by FACS analysis. N. Hematoxylin and eosin (HE) staining and immune histochemistry analyses of hsa-miR-12462 OE and MOCK tumor sections. HE staining magnification: ×20. MPO staining magnification: ×20. Ki-67 staining magnification: ×20.O-P. Percentage of myeloperoxidase (MPO) and Ki-67 expression in OE and MOCK U937 cells (MOCK vs. OE: O, P<0.0001. P, P<0.0001) [file 13045_2020_935_MOESM2_ESM.jpg]

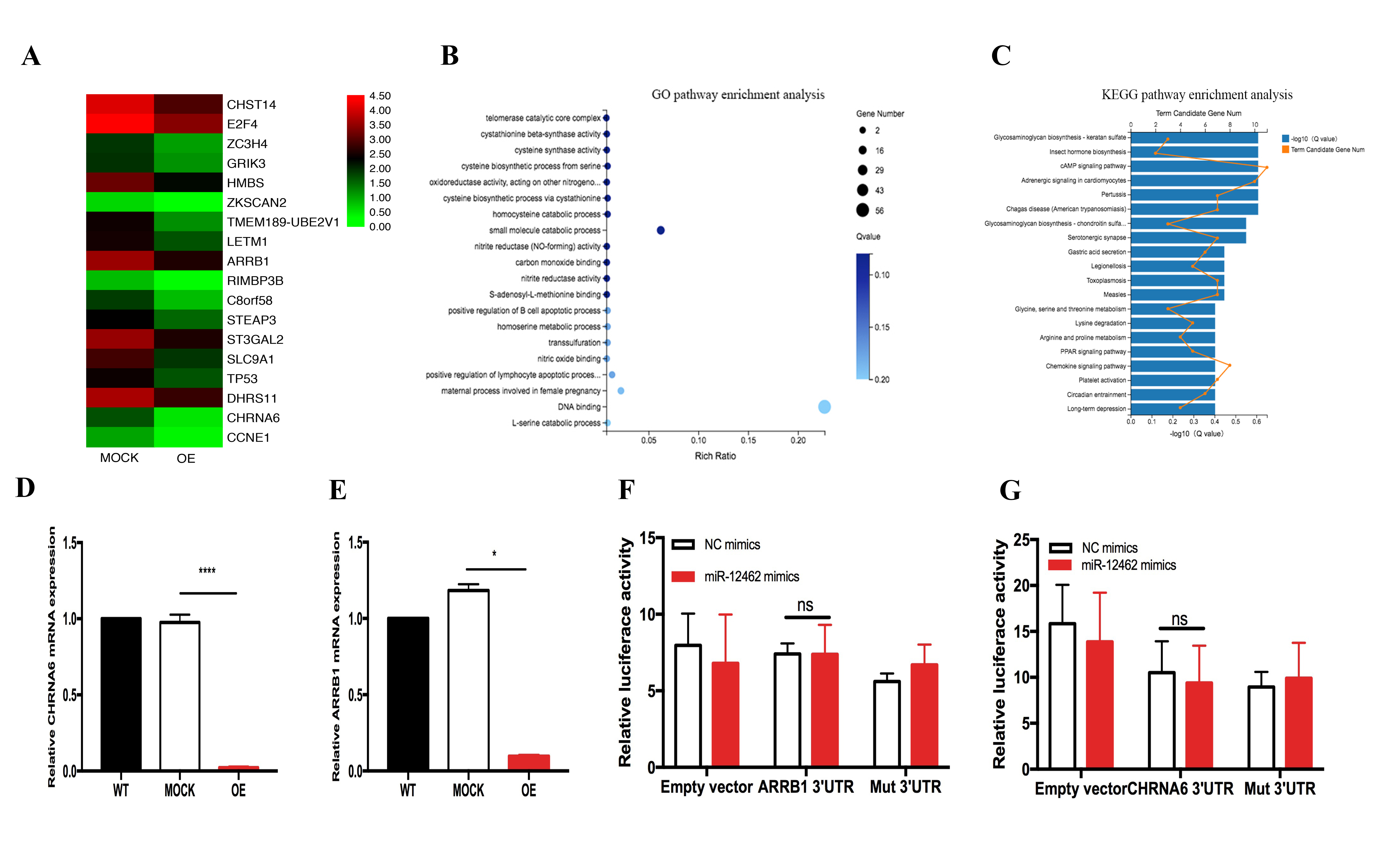

Supplement: Supplementary file 3 — Additional file 3: Figure S2. The downstream targets of hsa-miR-12462. A. Differential expression of selected genes in hsa-miR-12462 OE and MOCK U937cells. B-C. (B) GO and (C) KEGG pathway enrichment analysis of differentially expressed genes after RNA-sequencing in U937 OE/MOCK cells. D-E. Transcript expression of CHRNA6 (D) and ARRB1 (E) in wild-type, MOCK-infected and OE U937 cells is shown. F-G. Luciferase reporter assay showing the 3'-UTR segments of (F) ARRB1 and (G) CHRNA6 do not contain hsa-miR-12462 binding sites. Error bars indicate ± SD; n=3 each. P values were obtained by the two-tailed Student t-test. [file 13045_2020_935_MOESM3_ESM.jpg]
