# Supplementary material for: Roles of hsa-miR-12462 and SLC9A1 in acute myeloid leukemia
Source: J Hematol Oncol. 2020 Jul 23;13:101. doi: 10.1186/s13045-020-00935-w (PMC7376648; doi:10.1186/s13045-020-00935-w)
Supplement: Supplementary file 4 — Additional file 4. Detailed materials and methods [file 13045_2020_935_MOESM4_ESM.docx]

**Materials and methods**

***Cell culture and transfection***

U937 and HL-60 cells were obtained from the Cell Resource Centre (Xiangya Medical College, Central South University, Hunan, China). Cell lines were cultured in RPMI 1640 medium (Corning Inc., Corning, NY, USA) containing 10% foetal bovine serum (Corning Inc.) and 1% antibiotic solution of penicillin/streptomycin (Sigma, MO, USA) in a 37 °C incubator with a humidified atmosphere containing 5% CO_2_. To overexpress hsa-miR-12462, AML cells were infected with lentivirus (lentivirus vector: pGC-FU-3FLAG-SV40-EGFP-IRES-puromycin; promoter: Ubiqutin) containing the open reading frame of hsa-miR-12462 (MOI: 30). After 12 h of infection, we replaced the media for theU937 cell lines and continued to culture the cells for another 72 h. Then, the transfection efficiency was observed under a fluorescence microscope.

***Patient characteristics***

Bone marrow (BM) samples from 128 patients aged 15 to 71 years old who were diagnosed with AML according to the 2016 WHO criteria and treated in Xiangya Hospital of Central South University, Hunan, China, All subjects were grouped into 2 cohorts: (1) those achieving a complete remission (CR) with conventional induction chemotherapy and remaining in CR ≥ 6 months; and (2) those not achieving CR after 2 courses of standard induction chemotherapy (refractory) or relapsed in < 6 months after CR (relapsed). All the BM samples were collected into sterile tubes containing anticoagulant (heparin sodium). Mononuclear cells (MNCs) were enriched by density centrifugation with Ficoll-Paque (Sigma, St. Louis, MO, USA) and stored at −80 °C. The experimental protocols were approved by the ethics committee of Xiangya Hospital, Central South University. Informed consent was obtained from all the research subjects.

***qRT-PCR for miRNA and mRNA expression***

Quantitative real-time polymerase chain reaction (qRT-PCR) analysis for hsa-miR-12462 was performed in triplicate using miRNA First-strand cDNA synthesis (Takara) and SYBR® Green PCR Master Mix (Takara) according to the manufacturer's instructions. U6 snRNA levels were used for normalization. Primers for these miRNAs and U6 snRNA were obtained from RiboBio Corporation (Guangzhou, China). The fold change for each miRNA in leukaemic cells transfected with lentiviral vector (wild-type group relative to the negative control group) was calculated using the 2^-ΔΔCT^ method .

***Western blot analysis***

Equal amounts of protein were solubilized in sample buffer, electrophoresed on denaturing SDS-polyacrylamide gels and then transferred to polyvinylidene fluoride (PVDF) membranes (Millipore, Billerica, MA, USA). The membranes were saturated in TBST containing 5% BSA (Bio Sharp Sigma A-4612) for an hour at room temperature and then incubated with the primary antibodies overnight at 4 °C. After the blots were incubated with secondary antibody, they were washed three times, and protein bands were detected with a ChemiDoc MP System (Bio-Rad Laboratories. Inc., Hercules, CA, USA).

***Cell cycle analysis***

Cell cycle distribution was analysed using a cell cycle staining kit (Liankebio, China). As before, 1×10e^6^ cells were seeded in 6-well plates and incubated for 24 h. Cells were harvested, washed twice with 1×PBS, and incubated with 1 μl of DNA staining solution and 10 μl of permeabilization solution (PI) in the dark for 30 min at room temperature. The number of cells in each phase of the cell cycle was analysed using a flow cytometer (Becton Dickinson, CA, USA).

***EdU staining***

An EdU kit (RIBOBIO, Guangzhou, China) was used to measure DNA synthesis according to the manufacturer’s instructions. EdU is a thymidine analogue that can be incorporated into DNA during cell proliferation. Following the incorporation of EdU, a fluorescent molecule was added to react with EdU for fluorescent visualization of proliferating cells. All steps were carried out at room temperature.

***CCK8 assay***

Cell proliferation was measured with a Cell Counting Kit-8 assay (7sea biotech, China) using different concentrations of Ara-C (Solarbio, Lot. No. 317B002) according to the protocol provided by the manufacturer. Briefly, 2×10e^4^ cells were seeded in 96-well culture plates in 90 μL of medium per well and treated with different concentrations (0.25 μM, 0.5 μM, 1 μM, 2 μM, 4 μM, 8 μM, 16 μM, 32 μM, 64 μM) of Ara-C for 24 h and 48 h, respectively. Finally, 10 μl of CCK8 solution was added to each well, and the cells were incubated further for 3 h at 37 °C. Cell viability was evaluated based on the absorbance at 450 nm compared with that at 630 nm. Cell survival rate was calculated as follows: (%)=((experiment group-blank group)/(control group-blank group))*%.

***Annexin V-7-AAD assay***

A total of 1×10e^6^ cells were seeded in each well of 12-well culture plates and treated with Ara-C at its IC50. After 24 h, the cells were analysed by flow cytometry with an Annexin V-7-AAD kit (BD, USA) to determine the rate of cell apoptosis.

***Small RNA Sequencing***

Total RNA was extracted from the tissues using TRIzol agent according to the manual instructions for Small RNA TrueSequencing. Subsequently, total RNA was qualified and quantified using a NanoDrop and an Agilent 2100 bioanalyser. Total RNA was purified by electrophoretic separation on a 15% urea denaturing polyacrylamide gel electrophoresis (PAGE) gel, and regions corresponding to the 18–30 nt small RNA bands in the marker lane were excised and recovered. Then, the 18–30 nt small RNAs were ligated to a 5’-adaptor and a 3’-adaptor, which were subsequently transcribed into cDNA by SuperScript II Reverse Transcriptase (Invitrogen, USA), and then several rounds of PCR amplification with PCR Primer Cocktail and PCR Mix were performed to enrich the cDNA fragments. The PCR products were selected by agarose gel electrophoresis with target fragments of 100~120 bp and then purified with a QIAquick Gel Extraction Kit (QIAGEN, Valencia, CA). The final PCR ligation products were sequenced using the BGISEQ-500 platform (Small RNA TrueSeq, BGI-Shenzhen, China). Gene expression levels were measured in RPKM using Cufflinks.

***RNA-Sequencing***

RNA sample quality was analysed, and cDNA libraries were synthesized and sequenced using BGI technology. In brief, an Agilent 2100 Bioanalyser (Agilent) as used to assess the quality of the RNA samples and generate the cDNA libraries. Each library was sequenced on a HiSeq4000 (Illumina) using single reads. Gene expression levels were measured in RPKM using Cufflinks.

**In vivo *xenograft mice model***

The in vivo effect of hsa-miR-12462 on tumour growth was evaluated in a xenograft mouse model of AML. All animal protocols were performed in the animal facility at Central South University in accordance with federal, local, and institutional guidelines. Briefly, 0.1 ml of U937 cell suspension at a density of 5×10e^6^/ml was subcutaneously injected into the right dorsum of female nude mice (6 weeks of age, ~20 g). Tumour size was measured every 2 days starting from day 10 using a calliper, and tumour volume was calculated using the formula V = length × width^2^ × π / 6. The body weights of the mice were recorded under the same conditions. The mice were sacrificed at week 5, and the tumours were extracted.

***Magnetic resonance imaging***

Magnetic resonance imaging (MRI) was performed to analyse ectopically implanted tumours on nude mice at time points immediately before the mice were sacrificed. MRI was performed after mice were continuously anesthetized under 1–2% isoflurane. A clinical 3.0 T Siemens PRISMA MR scanner (Siemens, Erlangen, Germany) was used with a custom-designed small-animal-sized coil (Siemens, Erlangen, Germany). To obtain accurate and reproducible images, the mice were placed prone in animal tubes. The body temperature was maintained throughout the MRI studies with a warm blanket (37 °C). The imaging protocols were carried out in the axial coronal and sagittal plane with a field of view (FOV) of 6.4 cm, 256×192 pixels, slice thickness of 1.2 mm, and an in-plane resolution of 0.9×0.9 mm, and approximately 15-20 slices were acquired in each plane depending on the sizes of animals. The protocols were as follows: (a) pre-contrast T2-weighted fast-spin-echo (TR/TE 4000/85, ET 12); (b) pre-contrast T1-weighted spin-echo (TR/TE 400/15) coronal and transverse images; and (c) T1 mapping, which was performed using an SMRAT1Map sequence; non-selective IR; flip angle 50°; matrix 192x128; FOV 28 cm; TI 100 ms; thickness 8 mm; 3 inversions; and TI increment 100-150 ms, end-diastolic phase.

***Statistical analysis***

Each experiment was performed at least three times, and the data are represented as the mean ± SD of the vehicle controls. The groups of AML patients and donors or cell lines were compared using Statistical Package for the Social Sciences (SPSS) version 17.0, and a *P* value < 0.05 was considered statistically significant. The differences among these groups were tested by Student’s t-test or one-way ANOVA, as appropriate. Diagrams were drawn using GraphPad Prism 7 software.
